# Spatio-temporal variability in the distribution of ground-dwelling riparian spiders and their potential role in water-to-land energy transfer along Hong Kong forest streams

**DOI:** 10.7717/peerj.1134

**Published:** 2015-07-28

**Authors:** Elaine Y.L. Yuen, David Dudgeon

**Affiliations:** School of Biological Sciences, The University of Hong Kong, Hong Kong SAR, People’s Republic of China

**Keywords:** Tropical, Sparassidae, Lycosidae, Agelenidae, Suction sampling, Dietary reliance, Biomass

## Abstract

Terrestrial predators have been shown to aggregate along stream margins during periods when the emergence of adult aquatic insects is high. Such aggregation may be especially evident when terrestrial surroundings are relatively unproductive, and there are steep productivity gradients across riparia. In tropical forests, however, the productivity of inland terrestrial habitats may decrease the resource gradient across riparia, thus lessening any tendency of terrestrial predators to aggregate along stream margins. We elucidated the spatio-temporal variability in the distribution of ground-dwelling spiders and terrestrial arthropod prey within the riparia of two forest streams in tropical Hong Kong by sampling arthropods along transects at different distances from the streams during the wet and dry seasons. Environmental variables that may have influenced spider distributions were also measured. The vast majority of ground-dwelling predators along all transects at both sites were spiders. Of the three most abundant spiders captured along stream margins, *Heteropoda venatoria* (Sparassidae) and *Draconarius* spp. (Agelenidae) were terrestrially inclined and abundant during both seasons. Only *Pardosa sumatrana* (Lycosidae) showed some degree of aggregation at the stream banks, indicating a potential reliance on aquatic insect prey. Circumstantial evidence supports this notion, as *P. sumatrana* was virtually absent during the dry season when aquatic insect emergence was low. In general, forest-stream riparia in Hong Kong did not appear to be feeding hotspots for ground-dwelling predators. The lack of aggregation in ground-dwelling spiders in general may be attributed to the low rates of emergence of aquatic insects from the study streams compared to counterpart systems, as well as the potentially high availability of terrestrial insect prey in the surrounding forest. *Heteropoda venatoria*, the largest of the three spiders maintained a high biomass (up to 28 mg dry weight/m^2^) in stream riparia, exceeding the total standing stock of all other spiders by 2–80 times. The biomass and inland distribution of *H. venatoria* could make it a likely conduit for the stream-to-land transfer of energy.

## Introduction

Fluxes of aquatic insects from streams to the terrestrial landscape provide an important energy source for riparian insectivores, which may exhibit shifts in their spatio-temporal distribution in response to the availability of this water-to-land subsidy (Baxter, Fausch & Carl Saunders, 2005; Ballinger & Lake, 2006; Richardson & Sato, 2015). These shifts are indicative of consumer reliance on aquatic insects, and may have broader implications for riparian community composition that can include suppression of terrestrial prey populations (e.g., Murakami & Nakano, 2002; Sabo & Power, 2002) or changes in food web architecture (e.g., Henschel, Mahsberg & Stumpf, 2001).

Although the distributions of riparian insectivores are often responsive to the timing and magnitude of subsidies of emerging aquatic insects, the sensitivity of such responses are taxon-specific (e.g., Iwata, Nakano & Murakami, 2003; Sanzone et al., 2003; Paetzold, Bernet & Tockner, 2006). Such responses may be influenced by factors such as the ratio of subsidy to equivalent ambient resources (reviewed by Marczak, Thompson & Richardson, 2007) in the riparia, and the prey or microhabitat preferences of different insectivore taxa (e.g., Iwata, Nakano & Murakami, 2003; Greenwood & McIntosh, 2008; Hagen & Sabo, 2014).

The emergence of aquatic insects, and consequential water-to-land trophic subsidy, is likely to result in a steep productivity gradient across the riparian zones that leads to the aggregation of consumers, such as ground-dwelling insectivores, along stream margins; the aggregation can be especially evident in instances where rivers or streams are bordered by wide, relatively unproductive habitats such as gravel beds or sand bars (e.g., Sabo & Power, 2002; Sanzone et al., 2003; Paetzold, Schubert & Tockner, 2005). Small forest streams, with well-vegetated margins and productive habitat further inland, are likely to have less steep productivity gradients across riparia (Marczak & Richardson, 2007), thus reducing the tendency for insectivores to aggregate along streams. However, one of the few studies of volant insects that has been undertaken in tropical stream riparia showed that aquatic insects were largely confined to the margins of forest streams in Hong Kong (50–85% of total abundance within 10 m of the banks), whereas the distribution of volant terrestrial insects was unaffected by proximity to the stream (Chan, Zhang & Dudgeon, 2007). High densities of volant insects in these Hong Kong riparia made them potential feeding hotspots for birds, particularly during the wet season (Chan et al., 2008). Riparian web-building tetragnathid spiders also aggregated in and along the stream channel (Chan, Zhang & Dudgeon, 2009), and the same phenomenon has been reported for spiders along small streams draining temperate forests (Marczak & Richardson, 2007).

Due to their relatively limited mobility, ground-based predators are likely to be less sensitive to changes in the availability of aquatic insects than are flying insectivores (Power et al., 2004). In addition, they will feed on ground-dwelling terrestrial prey in addition to aquatic insects, and may respond to the aquatic subsidy differently from insectivores that specialize on volant prey (e.g., web-building spiders: Foelix, 2010). Studies of the distribution of ground-dwelling insectivores within riparia along small forest streams, as well as the potential availability of their non-aquatic prey, appear to be lacking thus far, and knowledge of the spatio-temporal dynamics of ground-dwelling predators in tropical stream riparia as a whole is generally poor (see reviews by Baxter, Fausch & Carl Saunders, 2005; Ballinger & Lake, 2006; Richardson & Sato, 2015).

The present study is part of a broader investigation intended to reveal the strength of water-to-land interactions mediated by arthropods along forest streams in tropical Hong Kong, southern China. The broader study involves: (1) seasonal patterns in the emergence rates of aquatic insects, and their relative contribution to total abundance of volant insects in riparia; (2) use of stable-isotope analysis to estimate the dietary dependence of predatory riparian arthropods on aquatic insects; and (3) the spatial and seasonal distribution of predatory ground-dwelling arthropods within stream riparia .

Spiders are pre-eminent among the ground-dwelling predators along forest streams in Hong Kong, southern China. Stable-isotope analysis of three common cursorial spiders within riparia along three forest streams in Hong Kong revealed that they all had significant dietary reliance (∼30–60%) on aquatic insects, although the degree of dependence varied somewhat among species (Yuen & Dudgeon, in press, see Table S1). Such variation could result from interspecific differences in spider phenology and life styles, or in their distribution and microhabitat preferences. Here we compare the spatio-temporal distribution of three genera of ground-dwelling spiders (Agelenidae, Lycosidae and Sparassidae) within riparia of two Hong Kong forest streams during the wet and dry seasons. We related these patterns to the abundance of potential prey, as well as environmental factors which may influence spiders. We expected that spiders would show some degree of aggregation along the stream banks, but that the extent of aggregation might depend on the relative availability of ground-dwelling prey and the species-specific characteristics of the spiders. The results of this investigation, in combination with data on spider dietary reliance on the aquatic subsidy, have implications for the roles of spiders in water-to-land energy transfer (e.g., Paetzold, Schubert & Tockner, 2005).

## Materials and Methods

### Study sites

**Figure 1:**
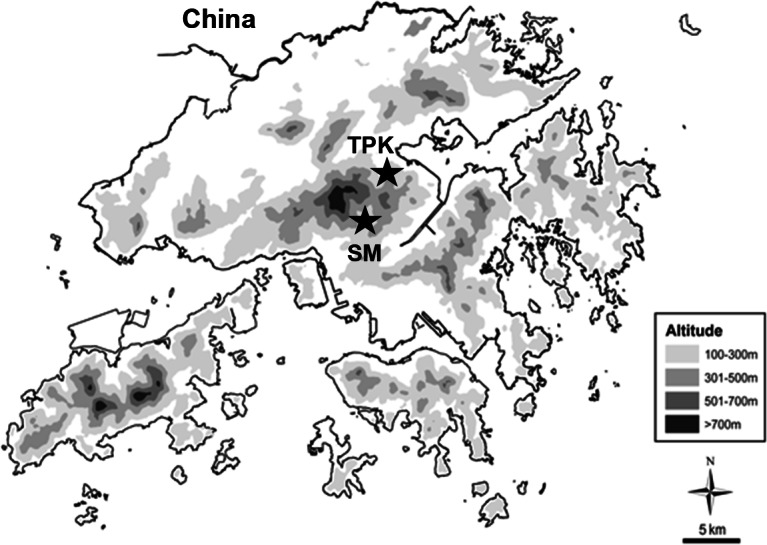
Locations of the two study reaches in Tai Po Kau Forest Stream (TPK) and Lead Mine Pass Stream (SM).

The study reaches were along Tai Po Kau Forest Stream (TPK: 3rd order; 22°25′24″N, 114°10′48″E) and along Lead Mine Pass Stream (SM: 4th order; 22°23′51″N, 114°09′05″E) in Hong Kong, southern China (Fig. 1). They were two of the three sites used in our concurrent investigation of spider stable-isotope signatures (Yuen & Dudgeon, in press). Both were unpolluted hill streams situated in drainage basins dominated by secondary forest (>60 years regrowth) within protected areas established in the 1970s (Dudgeon & Corlett, 2011). The streams were thus well protected from human disturbance. The study reaches were situated at ∼200 m a.s.l. and were similar in terms of wet width (∼8 m during the wet season; ∼3.5 m during the dry season), depth (riffles: ∼0.5 m during the wet season; 0.3 m during the dry season; pools >1 m during both seasons), streambed substrate type (dominated by cobbles in riffles, gravels in slow-flowing regions) and canopy coverage (>80%) (Table 1). Sampling was undertaken along one 50-m section of bank adjacent to each reach. At SM the eastern bank was selected as it was gently-sloping (<10° at 0–6 m from the stream and up to 15° further inland), comprising a 2–6 m-wide accumulation of gravel bordered by dense clumps of *Acorus gramineus* (Acoraceae) at the stream margin and secondary forest inland, where common trees included *Syzygium jambos* (Myrtaceae), *Schefflera heptaphylla* (Araliaceae), *Pavetta hongkongensis* (Rubiaceae). The western stream bank at TPK had a steeper gradient (<10°within 3 m of the stream, and up to 30° further inland), consisting of small, narrow gravel patches (2–10 m long, 1–3 m wide) and large boulders (up to 1 m in diameter) interspersed by secondary forest mainly consisting of *Aporusa dioica* (Euphorbiaceae), *Rhodoleia championi* (Hameamelidaceae) and *Garcinia oblongifolia* (Guttiferae).

### Field sampling

Ground-dwelling spiders were sampled along the bank of each reach during the 2013 wet season (9th July–2nd October) and the following dry season (28th Nov, 2013–17th Feb, 2014). Samples were collected on five rain-free nights, each separated by at least 14 days, during both seasons.

Measurements of variables that might influence spider numbers were also made on each sampling occasion: i.e., soil moisture, standing stock of leaf litter, and abundance of potential prey (Wise, 1995; Graham, Buddle & Spence, 2003; DeVito et al., 2004). In a recent meta-analysis, Muehlbauer et al. (2014) showed that stream ‘signatures,’ reflected in the contribution of aquatic insects to prey assimilated by terrestrial spiders and predaceous beetles or in the abundance of these predators, generally fell to 50% of that at the stream margin at a distance of only 1.2 m inland. Therefore, sampling was conducted along four parallel 50-m transects: one (at 0 m) was along the stream margins, while the other three were established 2, 5 and 10 m inland. This arrangement was chosen so as to reveal whether spiders showed a tendency to aggregate along the stream margins. Due to the high frequency of spates and the rocky terrain along the stream banks, deployment of pitfall traps was not feasible. Instead, suction sampling was employed to collect ground-dwelling spiders. A random-number table (created for each sampling date using StatTrek.com) was used to select five quadrats (area = 0.7 m × 0.7 m) along the 0-m transect and three quadrats of the same size along each of the other three transects. The additional quadrats along the 0-m transect reflected the more variable topography close to the stream margins. A suction sampler modified from a leaf-blower (Model 125BVx; Husqvarna, USA; see also Stewart & Wright, 1995) was used to collect leaf litter and all ground arthropods from within each quadrat into a 0.5 mm mesh bag attached to the distal end of the hose. During sampling, one investigator operated the sampler at full throttle, while an assistant removed large wood debris and cobbles from the ground in front of the inlet hose to avoid clogging the sampler. Individual samples were transported to the laboratory in plastic bags where they were frozen at −20 °C prior to processing. Immediately after each suction sample had been collected, a single soil sample (1 cm depth, ∼20–30 cm^3^) was collected with a spoon beside each quadrat, and stored in air-tight BD Falcon™ 50 mL centrifuge tubes (BD Biosciences, San Jose, California, USA) for subsequent gravimetric estimation of soil moisture. Permission to collection samples for this study within protected areas was granted by the Agriculture, Fisheries and Conservation Department, the Government of the Hong Kong Special Administrative Region (ref. no. of permit: AF GR CON 09/51).

### Laboratory processing

Suction samples were thawed at room temperature for 30 min. All invertebrates were hand-picked and identified to order under a stereomicroscope (Leica Wild M3C; Leica Microsystems, Wetzlar, Germany) at 10X magnification and counted. Coleopterans were further identified to families and categorized as predators or non-predators. Opiliones were assumed to be predators. Common spiders were identified to genus or species wherever possible, and they and other invertebrates were oven-dried at 60 °C for 48 h and weighed to the nearest 0.1 mg using an electronic balance (Model AUW220D; Shimadzu Corporation, Japan). Leaf litter dry mass from each quadrat was measured using the same procedure. Soil samples were weighed using an electronic balance, oven-dried at 60 °C for 48 h, and reweighed. Gravimetric soil moisture content (*θ*) was obtained from:

\begin{eqnarray*} \displaystyle \theta =\frac{{m}_{w e t}-{m}_{d r y}}{{m}_{d r y}}&&\displaystyle \end{eqnarray*}

where *m_wet_* = wet weight of soil sample, *m_dry_* = dry weight of soil sample.

### Data analysis

Data from each quadrat (*n* = 280) were treated as independent samples and used to model the occurrence and abundance of the three commonest spider species. Due to the high incidence of zero counts (>70% zeros), zero-altered models with Poisson (ZAP) or negative binomial (ZANB) distribution were employed for this analysis to account for overdispersion of data caused by the excessive zeroes (Zuur et al., 2009). The modelling consisted of two stages. First, a logistic model using presence/absence data was developed to model spider occurrence; second, zero-truncated Poisson or negative binomial models were used to model the abundance of spiders based on counts of spiders in non-zero quadrats, whereupon the best-fitting model of the two alternatives was identified. Since ZAP are nested within ZANB models, the choice of fitting a Poisson or negative biominal distribution to the count data was based on likelihood ratio tests (*α* = 0.05) which permit testing of their relative statistical fit (Zuur et al., 2009). The likelihood ratio tests were performed using full nested models that included season, site, distance from stream, soil moisture, litter dry weight, abundance and biomass of all terrestrial invertebrates as explanatory variables in both logistic and count models. For spider species which were aggregated at the stream margins, the abundance and biomass of those terrestrial taxa which had higher densities close to the water’s edge was included as additional covariates in the full models. Litter dry weight was log (*x* + 1)-transformed to improve homoscedasticity, but this transformation had no beneficial effect on counts of terrestrial invertebrates. Data from 0 and 2-m transects, and 5 and 10-m transects at a site were pooled respectively to ensure data sufficiency (number of counts >5 per category) for the development of logistic models (Zar, 1999). Best-fit zero-altered models were then selected based on Akaike’s information criterion (AIC; Akaike, 1973) by removing predictor variables from the full models with the chosen distribution until the model with the lowest AIC value was identified. Zero-altered models were developed by using pscl package ver. 1.4.6 (Jackman, 2014), and likelihood ratio tests were performed using lmtest package ver. 0.9–33 (Zeileis & Hothorn, 2002) for R ver. 3.0.3 (R Core Team, 2013).

## Results

### Distribution and composition of ground-dwelling arthropods

**Figure 2:**
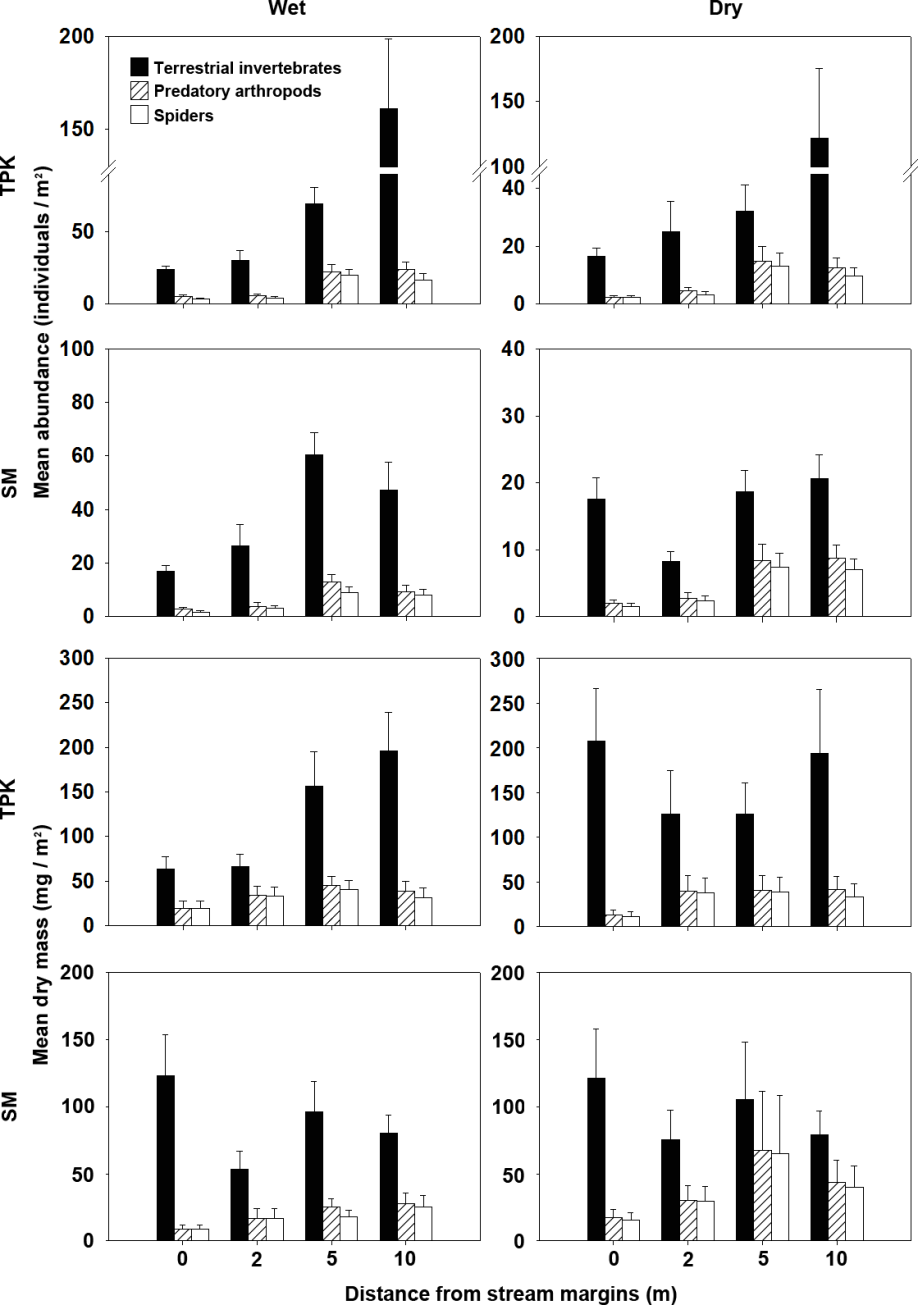
Mean abundance and drymass of terrestrial arthropods, predatory arthropods and spiders at four distances from the margins of the two study streams during thewet and dry seasons. Site abbreviations as in Fig. 1. Error bars, ±SEM. Note that values of abundance and dry mass at different sites and seasons are shown with different axes.

Arthropod abundance increased with distance from the stream margins at TPK and was generally similar between seasons (Fig. 2). Abundance at SM were generally higher along the 5 and 10-m transects during the wet season, and remained fairly constant with distance from the stream except being lower (∼50% of those along other transects) along the 2-m transect during the dry season (Fig. 2). Terrestrial invertebrate biomass also increased with distance from the stream during the wet season at TPK, but the same pattern was not observed during the dry season, when dry mass was along both the 0 and 10-m transects. At SM, mean biomass was highest along the 0-m transect and was similar during both seasons. Spiders were the most abundant predators at all transects at both sites, and made up 70–80% by number and 87–96% by biomass of all predators along the 0 and 2-m transects during the wet season and 70–95% by number and 88–100% by biomass during the dry season. Other predators found along the stream margins included beetles (mainly carabids and staphylinids, 0–28% by number), opiliones (0–18%), centipedes (0–5%) and pseudoscorpions (0–2%). Among the 24 orders of terrestrial invertebrates captured in this study, higher numbers and biomass of cockroaches and orthopterans (mainly gryllids and tetrigids) were found near the streams while other taxa were more abundant further inland (Tables S2 and S3).

### Spatial–temporal distribution of riparian spiders

**Figure 3:**
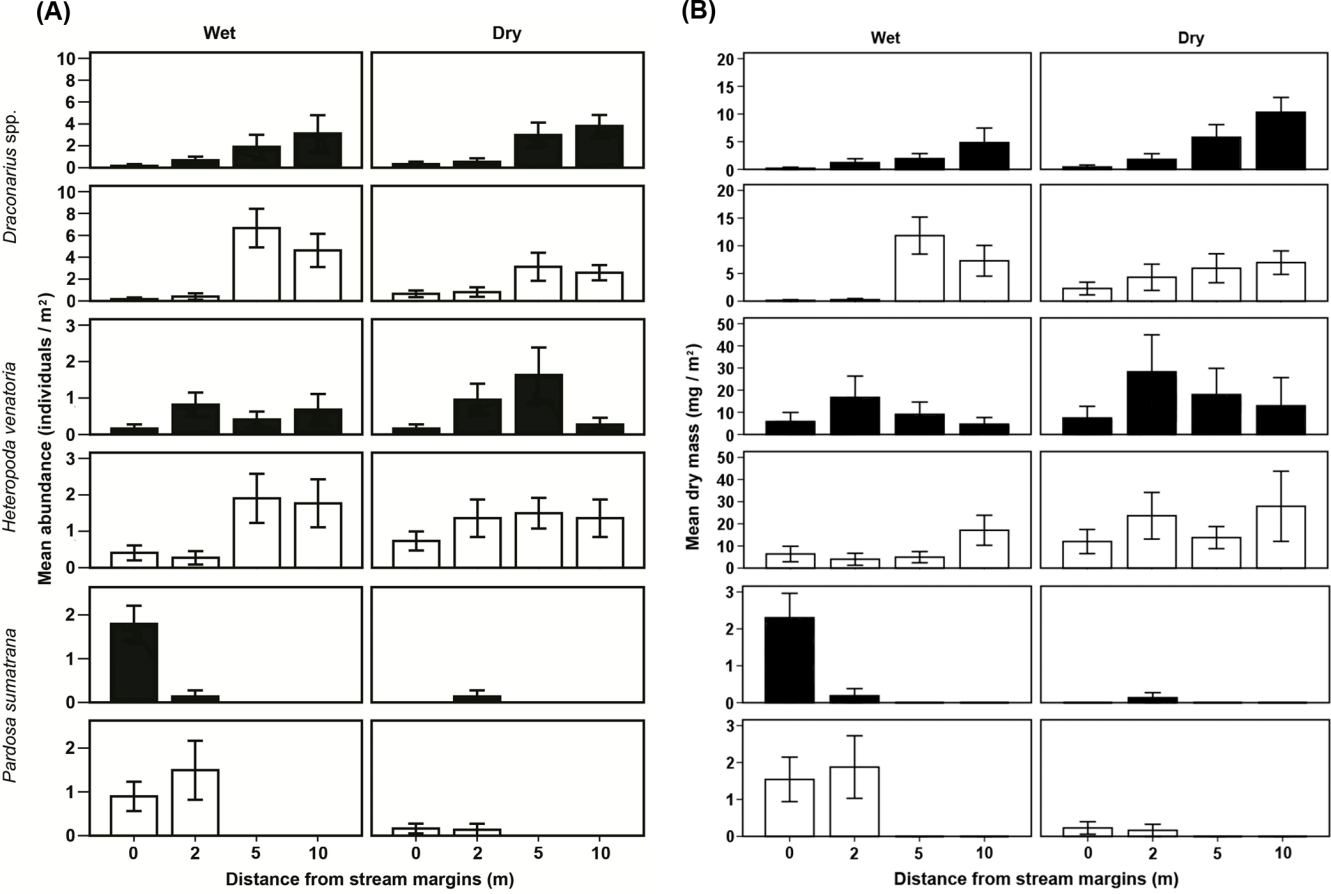
(A) Mean abundance and (B) mean dry mass of the three most abundant spiders at different distances from the margins of two streams during the wet and dry seasons. Filled bars, TPK; open bars, SM. Site abbreviations follow Fig. 1. Error bars, ±SEM. Note that the abundance scales and the dry mass scales differ between species and sites.

A total of 865 individuals of 11 families of ground-dwelling spiders were collected along the transects at SM and TPK, 21–26% of which were captured along the 0 and 2-m transects (Fig. 2). *Heteropoda venatoria* (Sparassidae; *n* = 113, 13.1% of all spiders collected), *Pardosa sumatrana* (Lycosidae; *n* = 49, 5.7%) and *Draconarius* spp. (Agelenidae; *n* = 246, 28.4%) were the most abundant spiders together constituting 65% of the total number and 77% of the total biomass of all spiders collected within 2 m of the streams. *Draconarius* spp. were scarce along the stream margins (only 6–25% in terms of number, and 3–39% in terms of biomass of overall captures, Figs. 3A and 3B) relative to the two more inland transects at both sites and during both seasons. *Heteropoda venatoria* occurred along all four transects at both sites during both seasons (Fig. 3A). At TPK, the abundance of this spider appeared to be evenly distributed across the riparian zone during the wet season, but tended to increasing with distance from the stream during the dry season (Fig. 3A), although biomass was highest 2m from the stream during both seasons (Fig. 3B). At SM, abundance and biomass data suggested that *H. venatoria* was terrestrially-inclined or evenly distributed across the riparia (Figs. 3A and 3B). At both sites, this species maintained the highest biomass (4–28 mg/m^2^, Fig. 3B) of any spider, exceeding the total standing stock of all other species by 2–80 times. *Pardosa sumatrana* was found only within 2 m of the stream at both sites; its abundance and biomass were more than 10 times higher during the wet season (Figs. 3A and 3B). The abundance and biomass of *P. sumatrana* was also 13 times higher along the 0-m than the 2-m transect at TPK during the wet season, but they were similar along both transects at SM (Figs. 3A and 3B). Lycosidae (15.1% of the total), Salticidae (7.1%), Amaurobiidae (6.8%), Theridiidae, (6.2%), Gnaphosidae (6.0%), Linyphiidae (4.2%), Oecobiidae (3.5%), Corinnidae (2%), Pisauridae (1%) and Sparassidae (1%) constituted the remainder of the spiders.

Zero-altered models were developed for *Draconarius* spp. and *H. venatoria* using data from both seasons as these were the only species for which sufficient count data were available from both sites and both seasons. *Pardosa sumatrana* appeared to aggregate along the stream margins, and thus the abundance and dry mass of cockroaches and orthopterans were included as additional covariates in the zero-altered models for this spider. Only wet-season data were used for this spider and distance was not included as an independent variable in the construction of zero-altered models, due to its very low abundance during the dry season and in areas inland. Likelihood ratio tests (*X*^2^ < 0.001, *p* > 0.9) suggested that the fit of ZANB models was not significantly different from ZAP models for *H.venatoria* and *P. sumatrana*, but was significantly better (*X*^2^ = 16.334, *p* < 0.001) than ZAP models for *Draconarius* spp. Accordingly, the results best-fit ZAP models are shown for *H. venatoria* and *P. sumatrana*, and the best-fit ZANB model for *Draconarius* spp.

The occurrence of *Draconarius* spp. was significantly higher at SM and positively related to the leaf-litter accumulation and terrestrial arthropod biomass in quadrats as shown by the best ZANB model (Fig. 3A and Table 2). In addition, both the occurrence and abundance of *Draconarius* spp. increased significantly with distance from the streams (Fig. 3A and Table 2).

**Table 1:** Site characteristics of stream sections at the two study sites during the wet and dry seasons. Site abbreviations as in Fig. 1.

Site		SM		TPK	
Season		Wet	Dry	Wet	Dry
Universal Transverse Mercator (UTM) grid reference		50Q KK 067 796		50Q KK 097 824	
Stream order		4th		3rd	
Flow regime		Perennial		Perennial	
Interval between the first sampling and the last flood		27 days		27 days	
Aspect		South-facing		North-facing	
Altitude of study reach (m)		200		190	
Canopy coverage at stream center		95%		89%	
Water depth (m)	Mean	0.33	0.33	0.56	0.25
	Range	0.19–0.58	0.19–0.58	0.39–0.86	0.18–0.30
Wet width (m)	Mean	3.2	3.2	8.4	3.9
	Range	1.5–4.0	1.5–4.0	6.0–10.5	1.4–6.8
Daily water temperature (°C)	Mean	15	15	24	16
	Range	12–21	12–21	23–26	12–24
Conductivity (μS cm^−1^)		38.0		43.5	
Dissolved oxygen (mg L^−1^)		8.3		8.8	
pH		6.6		6.8	
Ammonia N (μg L^−1^)		11.10		7.84	
Nitrite N (μg L^−1^)		1.61		1.79	
Nitrate N (μg L^−1^)		128.88		126.82	
Phosphate P (μg L^−1^)		1.37		8.01	

**Table 2:** Results of best-fit zero-altered models testing the effects of parameters on the distribution of the spiders *Draconarius* sp p., *H. venatoria* and *P. sumatrana*.

		Occurrence model				Abundance model			
Spider	Parameters	*γ*	SE	*z*-value	*P*	*B*	SE	*z*-value	*P*
*Draconarius* spp.^a^^,^^*^	Intercept	−3.168	0.832	−3.810	**<0.001**	0.174	0.222	0.785	0.432
	Site	1.078	0.341	3.160	**0.002**	–	–	–	**–**
	Distance	0.879	0.297	2.956	**0.003**	0.944	0.253	3.730	**<0.001**
	Leaf litter dry weight (log)	0.946	0.448	2.112	**0.034**	–	–	–	**–**
	Terrestrial arthropod abundance	–	–	–	**–**	0.004	0.002	1.880	0.060
	Terrestrial arthropod dry weight	4.416	1.821	2.425	**0.015**	–	–	–	**–**
*Heteropoda venatoria* ^a^ ^,^ ^*^	Intercept	−3.054	0.547	−5.579	**<0.001**	−0.624	0.364	−1.717	0.086
	Site	1.201	0.324	3.712	**<0.001**	–	–	–	–
	Distance	–	–	–	**–**	0.485	0.238	2.042	**0.041**
	Leaf litter dry weight (log)	0.897	0.275	3.263	**0.001**	–	–	–	–
	Terrestrial arthropod dry weight	2.794	1.669	1.674	0.094	–	–	–	–
*Pardosa sumatrana* ^b^ ^,^ ^**^	Intercept	2.479	0.898	2.761	**0.006**	1.637	1.019	1.606	0.108
	Soil moisture	−4.768	3.211	−1.485	0.138	–	–	–	–
	Leaf litter dry weight (log)	−2.745	0.902	−3.044	**0.002**	−2.044	1.443	−1.417	0.157
	Terrestrial arthropod abundance	–	–	–	**–**	0.038	0.017	2.228	**0.026**
	Orthopteran dry weight	63.333	26.590	2.382	**0.017**	−0.171	0.111	−1.539	0.124

**Notes.**

*γ* estimate of occurrence model *B* estimate of the abundance model SE standard error – term not included in the best-fit models

Significant *P*-values (*α* = 0.05) are in bold.

a Zero-altered model with negative binomial distribution (ZANB).

b Zero-altered model with Poisson distribution.

* Data from 0 and 2-m transects and data from 5 to 10 m transects were pooled within each site and each season to ensure number of non-zero-observations in each category were >5.

** Only data from the wet season were considered and data from all distance were pooled within each site.

The best-fit ZAP logistic model indicated that the occurrence of *H. venatoria* in quadrats increased significantly with the amount of leaf litter present, and was generally higher at SM (Fig. 3A and Table 2). The abundance of *H. venatoria* also increased with distance from the streams (Fig. 3A and Table 2).

The occurrence of *P. sumatrana* was negatively related to the amount of leaf litter but positively related to the biomass of Orthoptera (Table 2). The abundance of this spider was positively associated with terrestrial invertebrate abundance, although the relationship was weak (*B* = 0.038: Table 2).

## Discussion

Spiders made up the vast majority of ground-dwelling predators along all transects at both sites during each season. Distribution patterns across forest stream riparia in Hong Kong varied among the three most abundant spiders found near the stream margins: two of them (*Draconarius* spp. and *H. venatoria*) were terrestrially inclined, and only *P. sumatrana* showed some degree of aggregation at the stream banks.

Abundance of *Draconarius* spp., which capture prey by means of funnel webs (Wang, 2002), increased with distance from the streams. The zero-altered model indicated that their occurrence was positively related to the amounts of accumulated leaf litter. Dense vegetation and litter accumulations inland of the stream banks may modify air temperatures and prey availability which are known to affect habitat choice of funnel web spiders (Riechert & Gillespie, 1986). This notion gains support from the generally higher densities of ground-dwelling prey inland of the study streams, and the positive relationship between the occurrence and abundance of *Draconarius* spp. and prey density. Although data on the stable-isotope signatures of *Draconarius* spp., which are rather small (∼8 mm body length) and sparsely distributed, are not available, its predominately inland distribution suggests a low reliance on aquatic-insect prey. Furthermore, agelenids are generally disturbance-sensitive (Halaj, Halpern & Yi, 2008), probably due to the large amount of time and energy they need to invest in building funnel webs (Chen & Song, 1980; Tanaka, 1989). It may thus be possible that the distribution pattern in *Draconarius* spp. was also a result of avoidance of open areas adjacent to the channel which may be inundated during the frequent increases in water level associated with spates during the Hong Kong wet season.

The zero-altered model indicated that the distribution pattern of *H. venatoria* was consistent between seasons and its abundance increased with distance from the streams. Mean biomass of this species was found to be highest at 2 m from streams at TPK during both seasons, but this pattern was not observed in terms of abundance at this site or at SM. It is possible that larger individuals of *H. venatoria* aggregated at 2 m from TPK stream, but size data of individual spiders are needed to confirm this. Airamé & Sierwald (2000) found that spiders in this genus preferred microhabitats inland although they were sometimes encountered hunting along the stream bank. The occurrence of *H. venatoria* in Hong Kong was correlated with accumulations of leaf litter, but the genus is not confined to the ground and utilizes vertical surfaces of rocks and tree trunks (Airamé & Sierwald, 2000). Given that spiders may select substrates that match their body colors (e.g., Heiling et al., 2005; Fernández Campón, 2014), leaf litter may provide camouflage to *H. venatoria*, which has a brown or dark red body, rendering it cryptic.

*Pardosa sumatrana* was only found close to the stream, being confined to the 0 and 2-m transects at both sites. Restricted lateral dispersal of aquatic insects from streams, as reported in Hong Kong (Chan, Zhang & Dudgeon, 2007) and elsewhere (Muehlbauer et al., 2014), may cause spiders to concentrate foraging activities along the banks in order to access this water-to-land subsidy. This hypothesis is supported by the fact that spider stable-isotope signatures showed that the dietary reliance of *P. sumatrana* on aquatic insects was higher (by 7–14%) than that of *H. venatoria* which was distributed further inland (see Table S1; Yuen & Dudgeon, in press).

A limited tolerance of desiccation may also be one of the drivers of distribution in riparian lycosid spiders along gradients of soil moisture (Graham, Buddle & Spence, 2003; DeVito et al., 2004), but soil moisture was not identified as a limiting factor on the occurrence or abundance of *P. sumatrana* in the statistical model, nor was it uniformly higher close to the stream margins (EYL Yuen, 2014, unpublished data). *Pardosa sumatrana* occurrence was, however, negatively-related to leaf litter dry mass, so its association with the stream margins may reflect a preference for microhabitats with sparse vegetation or limited litter coverage. Indeed, this spider was mostly collected from exposed gravel patches along the stream banks. Many *Pardosa* spp. have highly particular microhabitat preferences (Lowrie, 1973; Moring & Stewart, 1994), and the restricted distribution of Hong Kong *P. sumatrana* may reflect an intrinsic species-specific substrate preference (e.g., Kraus & Morse, 2005). Alternatively, spiders may choose microhabitats in order to avoid predators (Rypstra et al., 2007) such as the much larger *H. venatoria* that occurred further inland and tended to be associated with leaf litter.

It was notable that the abundance of *P. sumatrana* was much lower (<10% of wet-season densities) during the dry season along both Hong Kong study streams. In temperate latitudes, juveniles of *Pardosa* spp. may hibernate during times when prey supply and temperature are low (Edgar, 1971; Edgar, 1972; Alderweireldt & Maelfait, 1988), whereas adults die after mating in spring or summer (Turnbull, 1966; Edgar, 1972; Buddle, 2000). Life-history information on tropical spiders is scant, and although the dry-season decrease of *P. sumatrana* occurred during a period of reduced abundance of terrestrial and aquatic insect prey (Yuen & Dudgeon, in press), there is no direct evidence of a causal link.

Overall, forest-stream riparia in Hong Kong did not appear to be feeding hotspots for ground-dwelling predators, unlike streams or rivers bordered by unproductive habitats (e.g., Sabo & Power, 2002; Sanzone et al., 2003; Paetzold, Schubert & Tockner, 2005). The emergence rates of aquatic insects from the study streams (0.2–4 mg DW m^−2^ day^−1^ in floating emergence traps during the 2013 wet season: Table S4) were much lower than reported for those systems (30–221 mg DW m^−2^ day^−1^ during the main emergence period: Sanzone et al., 2003; Paetzold, Schubert & Tockner, 2005) where ground-dwelling predators aggregate along stream margins. The lower availability of aquatic prey likely reduced reliance of Hong Kong spiders on this water-to-land energy, as shown by the fact that only *P. sumatrana* showed any degree of aggregation along the stream banks. The general pattern observed in these ground-dwelling spiders also contrasted with those of birds (Chan et al., 2008) and orb-weaving spiders (Chan, Zhang & Dudgeon, 2009) in Hong Kong, which are better adapted to exploiting on volant aquatic insects.

*Heteropoda venatoria* was the largest spider collected in the present study and maintained a high biomass (4–28 mg/m^2^), exceeding the total standing stock of other spiders by 2–80 times along all transects. Despite its lower dependence on aquatic insects (see Table S1; Yuen & Dudgeon, in press), this species would have exceeded all of the other spiders combined in terms of its potential importance in water-to-land transfer of energy, particularly given its tendency to occur both within the stream riparia (where most aquatic insects were concentrated: Chan, Zhang & Dudgeon, 2007) as well as further inland. Also, consumers may expand the influence of subsidies through their dispersal or movement (Soininen et al., 2015). However, thus far, we have no information on the individual home range of this spider, nor on how far the energy can be transferred inland through its movement. Mark-and-recapture studies combined with stable-isotope analysis of *H. venatoria* from inland could help to elucidate the amount and extent of the water-to-land energy transferred by this species.

## Supplemental Information

10.7717/peerj.1134/supp-1 Supplemental Information 1 Supplementary Information

10.7717/peerj.1134/supp-2 Data S1 Raw data

10.7717/peerj.1134/supp-3 Supplemental Information 2 Lead Mine Pass Stream (SM) in wet season 2013

10.7717/peerj.1134/supp-4 Supplemental Information 3 Tai Po Kau Forest Stream (TPK) in wet season 2013

10.7717/peerj.1134/supp-5 Supplemental Information 4 Permission for sample collection within protected areas

## Additional Information and Declarations

### Field Study Permissions

The following information was supplied relating to field study approvals (i.e., approving body and any reference numbers): Agriculture, Fisheries and Conservation Department, The Government of the Hong Kong Special Administrative Region, Permit No.: AF GR CON 09/51.

## References

[ref-1] Airamé S, Sierwald P (2000). Hunting and feeding behavior of one *Heteropoda* species in lowland rainforest on Borneo (Araneae, Sparassidae). Journal of Arachnology.

[ref-2] Akaike H, Petrov BN, Csaki F (1973). Information theory and an extension of the maximum likelihood principle.

[ref-3] Alderweireldt M, Maelfait J (1988). Life cycle, habitat choice and distribution of *Pardosa Amentata* (Clerck, 1757) in Belgium (Araneae, Lycosidae). Bulletin de la Societe Scientifique de Bretagne.

[ref-4] Ballinger A, Lake PS (2006). Energy and nutrient fluxes from rivers and streams into terrestrial food webs. Marine and Freshwater Research.

[ref-5] Baxter CV, Fausch KD, Carl Saunders W (2005). Tangled webs: reciprocal flows of invertebrate prey link streams and riparian zones. Freshwater Biology.

[ref-6] Buddle CM (2000). Life history of *Pardosa moesta* and *Pardosa mackenziana* (Araneae, Lycosidae) in Central Alberta, Canada. Journal of Arachnology.

[ref-7] Chan EK, Yu YT, Zhang Y, Dudgeon D (2008). Distribution patterns of birds and insect prey in a tropical riparian forest. Biotropica.

[ref-8] Chan EK, Zhang Y, Dudgeon D (2007). Contribution of adult aquatic insects to riparian prey availability along tropical forest streams. Marine and Freshwater Research.

[ref-9] Chan EK, Zhang Y, Dudgeon D (2009). Substrate availability may be more important than aquatic insect abundance in the distribution of riparian orb-web spiders in the tropics. Biotropica.

[ref-10] Chen Z, Song D (1980). The life style of *Agelena difficilis*. Chinese Journal of Zoology.

[ref-11] DeVito J, Meik JM, Gerson MM, Formanowicz DR (2004). Physiological tolerances of three sympatric riparian wolf spiders (Araneae: Lycosidae) correspond with microhabitat distributions. Canadian Journal of Zoology.

[ref-12] Dudgeon D, Corlett R (2011). The ecology and biodiversity of Hong Kong.

[ref-13] Edgar WD (1971). The life-cycle, abundance and seasonal movement of the wolf spider, *Lycosa* (*Pardosa*) *lugubris*, in Central Scotland. Journal of Animal Ecology.

[ref-14] Edgar WD (1972). The life-cycle of the wolf spider *Pardosa lugubris* in Holland. Journal of Zoology.

[ref-15] Fernández Campón F (2014). Substrate preference in a colonial spider: is substrate choice affected by color morph?. Entomological Science.

[ref-16] Foelix R (2010). Biology of spiders.

[ref-17] Graham AK, Buddle CM, Spence JR (2003). Habitat affinities of spiders living near a freshwater pond. Journal of Arachnology.

[ref-18] Greenwood MJ, McIntosh AR (2008). Flooding impacts on responses of a riparian consumer to cross-ecosystem subsidies. Ecology.

[ref-19] Hagen EM, Sabo JL (2014). Temporal variability in insectivorous bat activity along two desert streams with contrasting patterns of prey availability. Journal of Arid Environments.

[ref-20] Halaj J, Halpern CB, Yi H (2008). Responses of litter-dwelling spiders and carabid beetles to varying levels and patterns of green-tree retention. Forest Ecology and Management.

[ref-21] Heiling AM, Chittka L, Cheng K, Herberstein ME (2005). Colouration in crab spiders: substrate choice and prey attraction. The Journal of Experimental Biology.

[ref-22] Henschel JR, Mahsberg D, Stumpf H (2001). Allochthonous aquatic insects increase predation and decrease herbivory in river shore food webs. Oikos.

[ref-23] Iwata T, Nakano S, Murakami M (2003). Stream meanders increase insectivorous bird abundance in riparian deciduous forests. Ecography.

[ref-24] Jackman S (2014). pscl: classes and methods for R developed in the political science computational laboratory, Stanford University.

[ref-25] Kraus JM, Morse DH (2005). Seasonal habitat shift in an intertidal wolf spider: proximal cues associated with migration and substrate preference. Journal of Arachnology.

[ref-26] Lowrie DC (1973). The micro habitats of western wolf spiders of the genus *Pardosa*. Entomological News.

[ref-27] Marczak LB, Richardson JS (2007). Spiders and subsidies: results from the riparian zone of a coastal temperate rainforest. Journal of Animal Ecology.

[ref-28] Marczak LB, Thompson RM, Richardson JS (2007). Meta-analysis: trophic level, habitat, and productivity shape the food web effects of resource subsidies. Ecology.

[ref-29] Moring JB, Stewart KW (1994). Habitat partitioning by the wolf spider (Araneae, Lycosidae) guild in streamside and riparian vegetation zones of the Conejos River, Colorado. Journal of Arachnology.

[ref-30] Muehlbauer JD, Collins SF, Doyle MW, Tockner K (2014). How wide is a stream? Spatial extent of the potential “stream signature” in terrestrial food webs using meta-analysis. Ecology.

[ref-31] Murakami M, Nakano S (2002). Indirect effect of aquatic insect emergence on a terrestrial insect population through by birds predation. Ecology Letters.

[ref-32] Paetzold A, Bernet JF, Tockner K (2006). Consumer-specific responses to riverine subsidy pulses in a riparian arthropod assemblage. Freshwater Biology.

[ref-33] Paetzold A, Schubert CJ, Tockner K (2005). Aquatic-terrestrial linkages along a braided-river: riparian arthropods feeding on aquatic insects. Ecosystems.

[ref-34] Power ME, Rainey WE, Parker MS, Sabo JL, Polis GA, Power ME, Huxel GR (2004). River-to-watershed subsidies in an old-growth conifer forest. Food webs at the landscape level.

[ref-35] R Core Team (2013). R: a language and environment for statistical computing.

[ref-36] Richardson JS, Sato T (2015). Resource subsidy flows across freshwater–terrestrial boundaries and influence on processes linking adjacent ecosystems. Ecohydrology.

[ref-37] Riechert SE, Gillespie RG, Shear WA (1986). Habitat choice and utilization in web-building spiders. Spiders: webs, behavior and evolution.

[ref-38] Rypstra AL, Schmidt JM, Reif BD, DeVito J, Persons MH (2007). Tradeoffs involved in site selection and foraging in a wolf spider: effects of substrate structure and predation risk. Oikos.

[ref-39] Sabo JL, Power ME (2002). Numerical response of lizards to aquatic insects and short-term consequences for terrestrial prey. Ecology.

[ref-40] Sanzone DM, Meyer JL, Marti E, Gardiner EP, Tank JL, Grimm NB (2003). Carbon and nitrogen transfer from a desert stream to riparian predators. Oecologia.

[ref-41] Soininen J, Bartels P, Heino J, Luoto M, Hillebrand H (2015). Toward more integrated ecosystem research in aquatic and terrestrial environments. BioScience.

[ref-42] Stewart AJA, Wright AF (1995). A new inexpensive suction apparatus for sampling arthropods in grassland. Ecological Entomology.

[ref-43] Tanaka K (1989). Energetic cost of web construction and its effect on web relocation in the web-building spider *Agelena limbata*. Oecologia.

[ref-44] Turnbull AL (1966). A population of spiders and their potential prey in an overgrazed pasture in Eastern Ontario. Canadian Journal of Zoology.

[ref-45] Wang XP (2002). A generic-level revision of the spider subfamily Coelotinae (Araneae, Amaurobiidae). Bulletin of the American Museum of Natural History.

[ref-46] Wise DH (1995). Spiders in ecological webs.

[ref-47] Yuen YL, Dudgeon D (2015). Dietary dependence of predatory arthropods on volant aquatic insects in tropical stream riparia. Biotropica.

[ref-48] Zar JH (1999). Biostatistical analysis.

[ref-49] Zeileis A, Hothorn T (2002). Diagnostic checking in regression relationships. R News.

[ref-50] Zuur AF, Ieno EN, Walker NJ, Saveliev AA, Smith GM (2009). Mixed effects models and extensions in ecology with R.

