# Supplementary material for: Spatio-temporal variability in the distribution of ground-dwelling riparian spiders and their potential role in water-to-land energy transfer along Hong Kong forest streams
Source: PeerJ. 2015 Jul 28;3:e1134. doi: 10.7717/peerj.1134 (PMC4525688; doi:10.7717/peerj.1134)
Supplement: Supplemental Information 1 [file peerj-03-1134-s001.docx]

**Table S1**. Mean estimates (in percentages) of contribution (95% credibility intervals) of three prey types to the diets of three cursorial spiders as derived by Baysian mixing models (for details see Yuen & Dudgeon, 2015a). Spiders were collected during the wet and dry seasons from riparia of three forest streams in Hong Kong: Tai Po Kau Forest Stream (TPK), Lead Mine Pass Stream (SM) and Mui Tsz Lam Stream (MTL). *Hygropoda higenaga* was not present during the dry season.

|  |  | TPK | | SM | | MTL | |
| --- | --- | --- | --- | --- | --- | --- | --- |
| **Cursorial spider** | **Prey** | Wet | Dry | Wet | Dry | Wet | Dry |
| *Heteropoda venatoria* (Sparassidae) | Aquatic Insects | 32 (12-50) | 48 (30-67) | 37 (21-53) | 33 (15-50) | 34 (18-50) | 37 (21-53) |
|  | Terrestrial herbivores | 26 (6-45) | 21 (0-42) | 24 (6-42) | 32 (10-52) | 26 (6-44) | 26 (8-43) |
|  | Terrestrial detritivores | 42 (24-61) | 31 (10-50) | 39 (19-60) | 36 (14-57) | 39 (19-61) | 37 (20-55) |
| *Hygropoda higenaga* (Pisauridae) | Aquatic Insects | 36 (14-57) | - | 43 (25-61) | - | 46 (20-74) | - |
|  | Terrestrial herbivores | 24 (2-44) | - | 25 (5-45) | - | 23 (0-47) | - |
|  | Terrestrial detritivores | 40 (20-60) | - | 31 (9-52) | - | 31 (1-57) | - |
| *Pardosa sumatrana* (Lycosidae) | Aquatic Insects | 39 (16-62) | 59 (39-78) | 51 (31-71) | 41 (22-60) | 40 (23-57) | 44 (28-61) |
|  | Terrestrial herbivores | 25 (1-45) | 20 (0-42) | 22 (1-43) | 30 (4-52) | 24 (3-43) | 21 (3-39) |
|  | Terrestrial detritivores | 36 (14-58) | 21 (1-39) | 27 (3-49) | 30 (4-52) | 36 (13-59) | 35 (16-53) |

**Table S2** Mean abundance (±SEM; individuals/m^2^) of terrestrial invertebrates at different distances from stream margins at the two study sites during the wet and dry seasons. Site abbreviations follow Fig. 4.1. *Comprising Acarina, Amphipoda, Chilopoda, Coleoptera, Collembola, Dermaptera, Diplopoda, Hemiptera, non-formicid Hymenoptera, Lepidoptera, Mantodea, Microcoryphia, Opiliones, Phasmatodea, Pseudoscorpionida and Psocoptera.

|  | |  | **Wet** | | | | **Dry** | | | |
| --- | --- | --- | --- | --- | --- | --- | --- | --- | --- | --- |
| **Distance from stream margins (m)** | | | **0** | **2** | **5** | **10** | **0** | **2** | **5** | **10** |
| TPK | Araneae | | 3.3±0.6 | 4.2±0.7 | 19.9±4.2 | 16.3±4.6 | 2.3±0.5 | 3.3±1 | 13.1±4.7 | 9.7±2.8 |
|  | Blattodea | | 4.9±1.1 | 2±0.9 | 1.4±0.6 | 1.1±0.8 | 4.3±1.2 | 0.5±0.3 | 0.7±0.7 | 0.3±0.2 |
|  | Formicidae | | 3.8±0.9 | 7.6±4.0 | 15.2±4.1 | 102.3±34.3 | 2.1±0.5 | 3.1±1.1 | 20.5±6.9 | 13.9±7.2 |
|  | Isopoda | | 0.5±0.3 | 6.7±2.9 | 18.9±5.9 | 23.4±5.3 | 0.1±0.1 | 8.0±5.5 | 7.6±2.0 | 12.1±4.4 |
|  | Isoptera | | 0.1±0.1 | 2.6±1.3 | 4.8±3.2 | 3.0±2.3 | 0±0 | 0±0 | 1.0±1.0 | 2.4±1.5 |
|  | Orthoptera | | 7.8±1.1 | 3.4±1.4 | 2.4±0.6 | 3.1±0.9 | 4.5±0.8 | 0.8±0.6 | 0.8±0.3 | 0.5±0.2 |
|  | Others* | | 3.3±0.9 | 4.2±1.2 | 7.3±1.4 | 11.8±3.2 | 3.9±0.6 | 2.3±0.8 | 13.6±2.7 | 6.9±2.4 |
|  | All terrestrial arthropods | | 23.8±2.2 | 30.1±7.1 | 69.4±11.3 | 161.1±37.6 | 16.5±2.8 | 25±10.4 | 32.1±9 | 121.6±53.7 |
| SM | Araneae | | 1.7±0.5 | 3±1 | 9±2 | 7.9±2.2 | 1.6±0.4 | 2.3±0.8 | 7.3±2.2 | 7.1±1.5 |
|  | Blattodea | | 5.7±1.4 | 5.7±1.5 | 1.5±0.7 | 0.1±0.1 | 4.2±1.1 | 4.1±1.4 | 1.8±0.8 | 0.5±0.2 |
|  | Formicidae | | 0.2±0.2 | 3.9±3.4 | 4.6±3.5 | 25.3±20.0 | 0.7±0.3 | 0.4±0.2 | 2.3±0.6 | 1.0±0.4 |
|  | Isopoda | | 0±0 | 0.8±0.3 | 3.9±1.4 | 4.1±1.5 | 0.6±0.3 | 0±0 | 1.1±0.7 | 1.6±0.6 |
|  | Isoptera | | 0±0 | 7.6±7.6 | 0±0 | 73.6±52.4 | 0±0 | 0.1±0.1 | 0.8±0.6 | 2.6±1.7 |
|  | Orthoptera | | 3.3±1.2 | 3.8±1.1 | 7.1±1.6 | 4.4±1.7 | 4.1±1.2 | 0.4±0.3 | 0.8±0.3 | 1.9±0.5 |
|  | Others* | | 5.0±1.8 | 7.9±3.9 | 9.0±3.9 | 8.2±3.9 | 6.9±3.1 | 1.0±0.3 | 4.4±0.9 | 6.0±1.9 |
|  | All terrestrial arthropods | | 16.8±2.1 | 26.5±7.9 | 60.3±8.3 | 47.2±10.4 | 17.6±3.3 | 8.3±1.4 | 18.6±3.3 | 20.7±3.6 |

**Table S3** Mean dry mass (±SEM; mg/m^2^) of terrestrial invertebrates at different distances from stream margins at the two study sites during the wet and dry seasons. Site abbreviations follow Fig. 4.1. *Comprising Acarina, Amphipoda, Chilopoda, Coleoptera, Collembola, Dermaptera, Diplopoda, Hemiptera, non-formicid Hymenoptera, Lepidoptera, Mantodea, Microcoryphia, Opiliones, Phasmatodea, Pseudoscorpionida and Psocoptera.

|  | |  | **Wet** | | | | **Dry** | | | |
| --- | --- | --- | --- | --- | --- | --- | --- | --- | --- | --- |
| **Distance from stream margins (m)** | | | **0** | **2** | **5** | **10** | **0** | **2** | **5** | **10** |
| TPK | Araneae | | 19.2±8.6 | 33.2±10.3 | 41±9.6 | 31.1±11.2 | 11.4±5.2 | 37.5±17 | 39.2±16 | 33.7±14.2 |
|  | Blattodea | | 27.8±12.8 | 1.4±0.8 | 4.1±3.1 | 2.8±2.1 | 125.9±40.4 | 17.4±15.3 | 6.1±6.1 | 2.4±1.6 |
|  | Formicidae | | 5±2 | 5.3±2.5 | 22.4±10.5 | 53.1±25.1 | 0±0 | 3.4±2.7 | 2.2±1.2 | 4.2±2.3 |
|  | Isopoda | | 1.1±0.8 | 14.7±7.1 | 41.5±14 | 66.2±16.3 | 0±0 | 2.7±1.5 | 18.2±5.8 | 14.5±5.9 |
|  | Isoptera | | 0.3±0.3 | 4.2±2.6 | 2.5±1.2 | 2.6±2 | 0±0 | 9.1±9.1 | 0±0 | 93.3±68.7 |
|  | Orthoptera | | 8.8±1.9 | 3.7±2.4 | 5±1.9 | 2.8±0.9 | 19.9±4.2 | 4.9±3.2 | 5.5±2.3 | 1.9±1.1 |
|  | Others* | | 1.4±0.6 | 4.3±1.1 | 40.1±24.5 | 37.3±12.8 | 50.5±39.1 | 50.7±27.5 | 63.7±20 | 43.9±13.2 |
|  | All terrestrial arthropods | | 63.7±13.9 | 66.7±13.6 | 156.6±38.5 | 196±43.3 | 207.7±58.9 | 125.7±49.4 | 126±34.5 | 194±71.5 |
| SM | Araneae | | 8.5±3.5 | 16.4±7.6 | 17.9±4.7 | 25.3±8.5 | 15.3±5.8 | 29.5±10.8 | 64.8±43.8 | 39.8±15.9 |
|  | Blattodea | | 100.8±28.8 | 6.8±1.7 | 1.7±0.8 | 0.1±0.1 | 78.6±36.1 | 19.9±6.8 | 6.3±3.4 | 4.3±2.2 |
|  | Formicidae | | 3.4±1.1 | 5±2.8 | 18.1±4.9 | 16.8±3.4 | 1.6±0.9 | 1.1±0.9 | 8±3.6 | 4.7±3 |
|  | Isopoda | | 0.4±0.4 | 4.2±3.8 | 13.8±5 | 20.6±8.7 | 1.4±0.8 | 0±0 | 2.6±2 | 4.4±3 |
|  | Isoptera | | 0±0 | 4.1±3.7 | 0.6±0.6 | 2.6±2.1 | 0±0 | 1.1±1.1 | 1.4±1.2 | 2.7±2 |
|  | Orthoptera | | 7.4±3.3 | 15.2±6.1 | 16.1±9.5 | 5.7±2.9 | 19.8±8.1 | 4.4±4.1 | 6.3±2.9 | 12.4±4.5 |
|  | Others* | | 2.8±0.9 | 1.7±0.8 | 27.9±8.6 | 8.9±2.1 | 4.8±1.7 | 19.2±17.7 | 15.2±6.4 | 11±3.6 |
|  | All terrestrial arthropods | | 123.4±30.3 | 53.3±13.5 | 96.3±22.7 | 80±13.8 | 121.4±36.8 | 75.1±22.1 | 105.2±43.1 | 79.1±17.4 |

**Table S4**. Means and ranges of average daily emergence rates (in mg DW/m^2^) in floating emergence traps at the two study sites during wet (May – October 2013) and dry (November 2013 – March 2014) seasons. Six floating emergence traps (mesh size 0.3 mm) with basal dimensions of 50 cm x 50 cm and a height of 25 cm (*cf.* Paetzold and Tockner 2005) were deployed each month, with each separated by at least 10 m and up to six days at both sites. Site abbreviations as in Fig. 1.

|  | **Wet** | **Dry** |
| --- | --- | --- |
| **TPK** | 1.3 (0.6-2.8) | 1.1 (0-2.0) |
| **SM** | 1.1 (0.2-4.0) | 0.7 (0-1.6) |

**Table S5**. Isotopic signatures (mean ± 1 SD) of riparian spiders and their potential prey at the two study sites during the wet and dry seasons. Only mean value was shown when the number of replicates was fewer than three. Site abbreviations as in Fig. 1.

| **Taxa group** | **Order / Infraorder** | **Family** | **Species** |  | **Wet** | | **Dry** | | |  |
| --- | --- | --- | --- | --- | --- | --- | --- | --- | --- | --- |
|  |  |  |  |  | **TPK** | **SM** | **TPK** | **SM** |  |  |
| **Predator** | **Araneae** | **Lycosidae** | ***Pardosa sumatrana*** | δ^13^C | -26.4 ± 0.5 | -26.4 ± 0.3 | -27.0 ± 0.4 | -26.5 ± 0.9 |  |  |
|  |  |  |  | δ^15^N | 4.7 ± 0.6 | 5.2 ± 0.6 | 5.1 ± 0.6 | 4.9 ± 1.1 |  |  |
| **Predator** | **Araneae** | **Sparassidae** | ***Heteropoda venatoria*** | δ^13^C | -27.3 ± 0.1 | -27.1 ± 0.3 | -26.9 ± 0.3 | -27.0 ± 0.3 |  |  |
|  |  |  |  | δ^15^N | 3.6 ± 0.7 | 3.9 ± 0.5 | 4.3 ± 0.5 | 4.6 ± 0.7 |  |  |
|  |  |  |  | δ^15^N | 4.8 ± 0.8 | 4.8 ± 0.5 | - | - |  |  |
| **Adult Aquatic Insect** | **Diptera** | **Chironomidae** | ***-*** | δ^13^C | -27.1 ± 0.3 | -27.2 ± 0.3 | -29.0 ± 0.3 | -27.8 ± 1.2 |  |  |
|  |  |  |  | δ^15^N | 4.0 ± 0.8 | 4.4 ± 0.6 | 3.1± 0.2 | 3.3 ± 0.4 |  |  |
| **Adult Aquatic Insect** | **Ephemeroptera** | **Baetidae** | ***-*** | δ^13^C | -27.8 | - | - | - |  |  |
|  |  |  |  | δ^15^N | 2 | - | - | - |  |  |
| **Adult Aquatic Insect** | **Ephemeroptera** | **Ephemeridae** | ***-*** | δ^13^C | -26.6 | -27.0 ± 0.2 | - | - |  |  |
|  |  |  |  | δ^15^N | 2.4 | 3.6 ± 0.2 | - | - |  |  |
| **Adult Aquatic Insect** | **Ephemeroptera** | **Heptageniidae** | ***-*** | δ^13^C | -30.1 | -23.4 ± 1.3 | - | - |  |  |
|  |  |  |  | δ^15^N | 3.5 | 2.8 ± 0.12 | - | - |  |  |
| **Adult Aquatic Insect** | **Ephemeroptera** | **Leptophlebiidae** | ***-*** | δ^13^C | -28.2 ± 0.4 | -27.6 ± 0.5 | - | - |  |  |
|  |  |  |  | δ^15^N | 3.1 ± 0.1 | 2.3 ± 0.6 | - | - |  |  |
| **Adult Aquatic Insect** | **Trichoptera** | **Glossosomatidae** | ***-*** | δ^13^C | -28.5 ± 0.3 | -26.3 ± 1.4 | -28.8 ± 1.1 | -25.3 ± 1.4 |  |  |
|  |  |  |  | δ^15^N | 1.0 ± 0.2 | 1.2 ± 0.2 | 1.7 ± 0.1 | 1.1 ± 0.1 |  |  |
| **Adult Aquatic Insect** | **Trichoptera** | **Hydropsychidae** | ***-*** | δ^13^C | -28.2 ± 0.4 | -27.3 ± 0.6 | - | - |  |  |
|  |  |  |  | δ^15^N | 3.9 ± 0.1 | 3.3 ± 0.1 | - | - |  |  |
| **Adult Aquatic Insect** | **Trichoptera** | **Hydroptilidae** | ***-*** | δ^13^C | -26.8 | - | - | - |  |  |
|  |  |  |  | δ^15^N | 3.7 | - | - | - |  |  |
| **Adult Aquatic Insect** | **Trichoptera** | **Odontoceridae** | ***-*** | δ^13^C | -24.7 | - | - | - |  |  |
|  |  |  |  | δ^15^N | 3.1 | - | - | - |  |  |
| **Adult Aquatic Insect** | **Trichoptera** | **Philopotamidae** | ***-*** | δ^13^C | -27.8 ± 0.3 | -27.8 ± 0.3 | -28.0 ± 0.4 | -27.7 |  |  |
|  |  |  |  | δ^15^N | 4.0 ± 0.1 | 3.3 ± 0.2 | 3.6 ± 0 | 3.9 |  |  |
| **Adult Aquatic Insect** | **Trichoptera** | **Psychomyiidae** | ***-*** | δ^13^C | - | -20.8 | - | -21.3 |  |  |
|  |  |  |  | δ^15^N | - | 2.7 | - | 2.8 |  |  |
| **Terrestrial Detritivores** | **Blattodea** | **Blaberidae** | ***-*** | δ^13^C | -27.7 ± 0.1 | -28.1 ± 0.2 | -28.5 ± 0.7 | -27.9 ± 0.8 |  |  |
|  |  |  |  | δ^15^N | -0.9 ± 0.1 | -0.4 ± 0.2 | -0.9 ± 0.17 | -0.5 ± 0.7 |  |  |
|  |  |  |  |  |  |  |  |  |  |  |
| **Terrestrial Detritivores** | **Isopoda** | **-** | ***-*** | δ^13^C | -27.1 ± 0.3 | -27.7 ± 0.3 | - | -27.9 ± 0.7 |  |  |
|  |  |  |  | δ^15^N | 0.6 ± 0.5 | 0.1 ± 0.2 | - | 0.8 ± 0.8 |  |  |
| **Terrestrial Detritivores** | **Isoptera** | **-** | ***-*** | δ^13^C | -26.1 ± 0.1 | -27.1 ± 0.1 | -28.7 ± 1.6 | - |  |  |
|  |  |  |  | δ^15^N | -1.4 ± 0.0 | -2.2 ± 0.2 | -2.5 ± 0.2 | - |  |  |
| **Terrestrial Herbivores** | **Coleoptera** | **Cerambycidae** | ***-*** | δ^13^C | -26.4 | - | - | - |  |  |
|  |  |  |  | δ^15^N | 2.3 | - | - | - |  |  |
| **Terrestrial Herbivores** | **Coleoptera** | **Chrysomelidae** | ***-*** | δ^13^C | -25.9 | - | - | - |  |  |
|  |  |  |  | δ^15^N | 2.5 | - | - | - |  |  |
| **Terrestrial Herbivores** | **Coleoptera** | **Scarabaeidae** | ***-*** | δ^13^C | -27.9 ± 0.3 | - | - | - |  |  |
|  |  |  |  | δ^15^N | 4.8 ± 0.9 | - | - | - |  |  |
| **Terrestrial Herbivores** | **Hemiptera** | **Cicadellidae** | ***-*** | δ^13^C | -30.1 | - | - | - |  |  |
|  |  |  |  | δ^15^N | -3.5 | - | - | - |  |  |
| **Terrestrial Herbivores** | **Hemiptera** | **Fulgoridae** | ***-*** | δ^13^C | - | -29.0 ± 0.5 | -29.9 ± 0.6 | - |  |  |
|  |  |  |  | δ^15^N | - | -0.6 ± 0.5 | -0.8 ± 0.2 | - |  |  |
| **Terrestrial Herbivores** | **Hemiptera** | **Lygaeidae** | ***-*** | δ^13^C | - | - | -28.6 ± 0.7 | - |  |  |
|  |  |  |  | δ^15^N | - | - | -1.7 ± 1.8 | - |  |  |
| **Terrestrial Herbivores** | **Hemiptera** | **Psylloidae** | ***-*** | δ^13^C | - | - | - | - |  |  |
|  |  |  |  | δ^15^N | - | - | - | - |  |  |
| **Terrestrial Herbivores** | **Hemiptera** | **Pyrrhocoridae** | ***-*** | δ^13^C | - | - | - | - |  |  |
|  |  |  |  | δ^15^N | - | - | - | - |  |  |
| **Terrestrial Herbivores** | **Lepidoptera** | **-** | ***-*** | δ^13^C | -30.5 ± 1.8 | -26.8 ± 1.4 | -31.2 ± 0.3 | -30.1 ± 1.1 |  |  |
|  |  |  |  | δ^15^N | 2.8 ± 1.2 | 2.2 ± 0.3 | 3.6 ± 1.0 | 1.6 ± 0.8 |  |  |
|  |  |  |  |  |  |  |  |  |  |  |
| **Terrestrial Herbivores** | **Orthoptera** | **Gryllacrididae** | ***-*** | δ^13^C | - | -27.8 | - | - |  |  |
|  |  |  |  | δ^15^N | - | 1.1 | - | - |  |  |
| **Terrestrial Herbivores** | **Orthoptera** | **Gryllidae** | ***-*** | δ^13^C | -28.7 ± 0.6 | -27.2 ± 0.2 | -28.1 ± 0.3 | -27.9 ± 0.3 |  |  |
|  |  |  |  | δ^15^N | 1.8 ± 1.2 | 1.9 ± 0.2 | 2.3 ± 0.3 | 1.9 ± 0.7 |  |  |
| **Terrestrial Herbivores** | **Orthoptera** | **Tetrigidae** | ***-*** | δ^13^C | -27.6 ± 1.1 | -26.4 ± 0.7 | -27.9 ± 0.5 | -26.1 ± 0.8 |  |  |
|  |  |  |  | δ^15^N | -1.6 ± 0.4 | -0.7 ± 0.4 | -1.3 ± 0.4 | -0.5 ± 0.4 |  |  |
