# Supplementary figures and images for: Spatio-temporal variability in the distribution of ground-dwelling riparian spiders and their potential role in water-to-land energy transfer along Hong Kong forest streams
Source: PeerJ. 2015 Jul 28;3:e1134. doi: 10.7717/peerj.1134 (PMC4525688; doi:10.7717/peerj.1134)

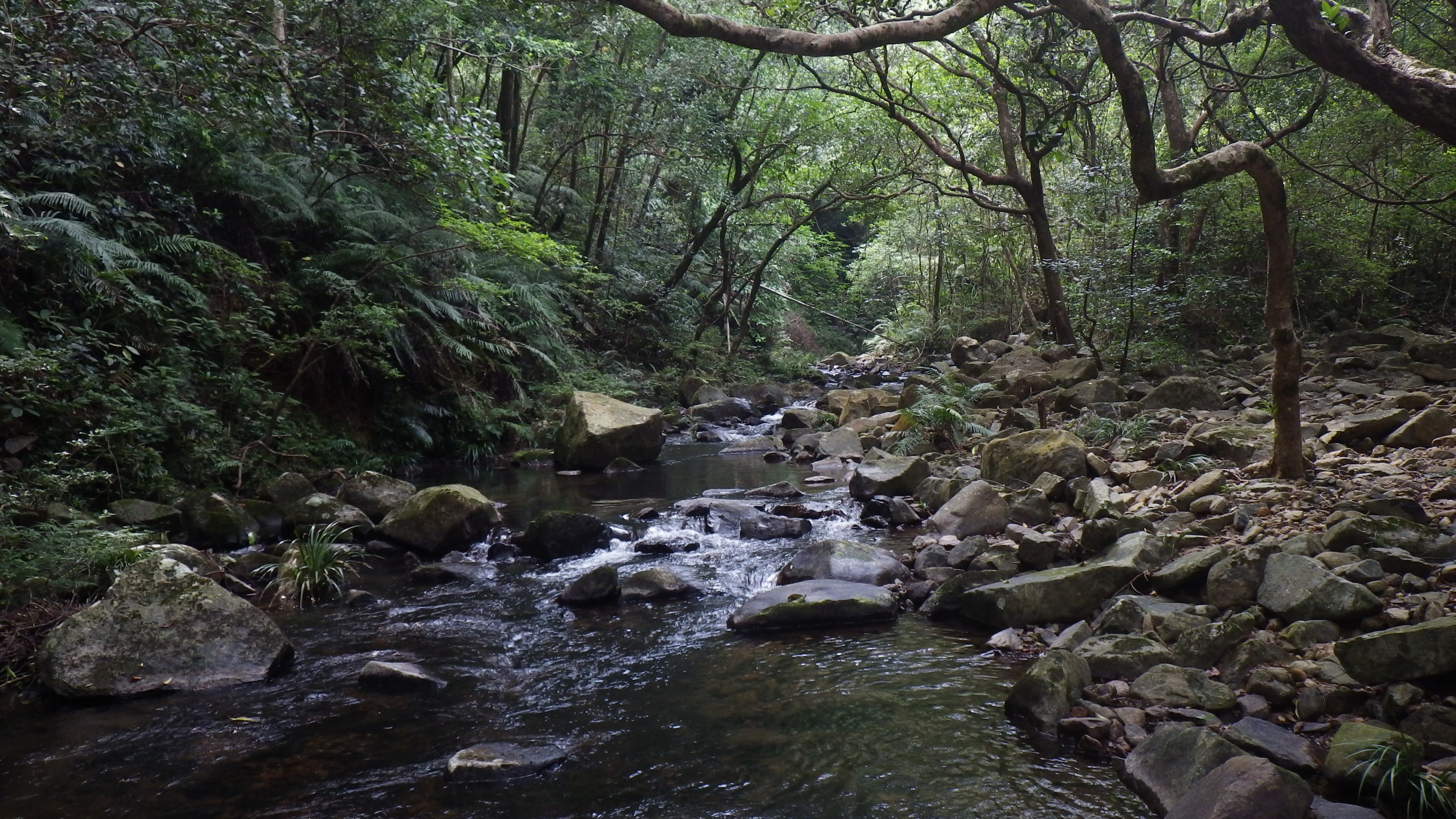

Supplement: Supplemental Information 2 [file peerj-03-1134-s003.jpg]

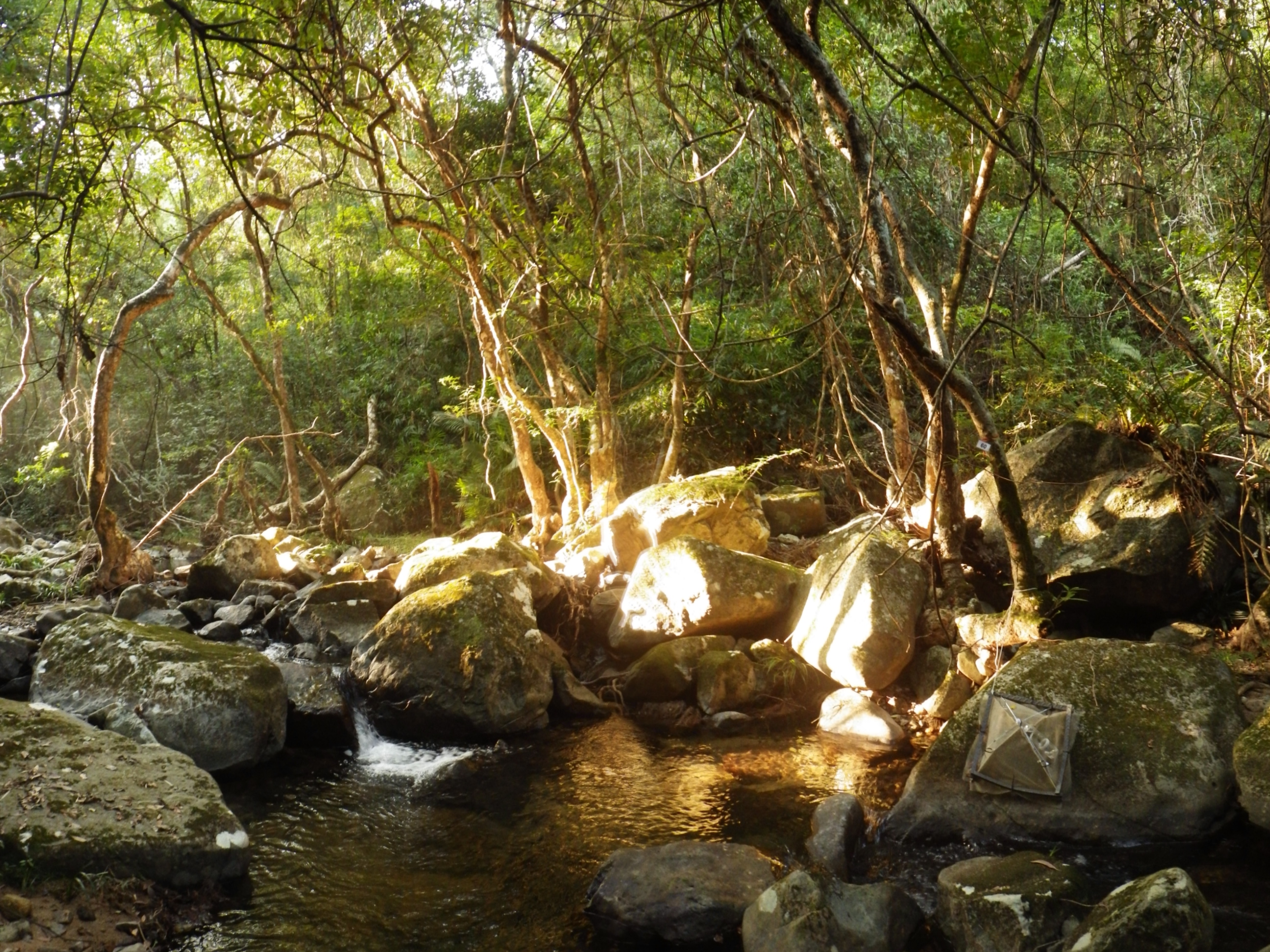

Supplement: Supplemental Information 3 [file peerj-03-1134-s004.jpg]
