# Supplementary material for: Spatio-temporal variability in the distribution of ground-dwelling riparian spiders and their potential role in water-to-land energy transfer along Hong Kong forest streams
Source: PeerJ. 2015 Jul 28;3:e1134. doi: 10.7717/peerj.1134 (PMC4525688; doi:10.7717/peerj.1134)
Supplement: Supplemental Information 4 [file peerj-03-1134-s005.pdf]

漁農自然護理署
九龍長沙灣道三O三號
長沙灣政府合署五樓

覆函請寄交
「漁農自然護理署署長」
Please address all replies to
Director of Agriculture, Fisheries and Conservation

**AGRICULTURE, FISHERIES AND CONSERVATION DEPARTMENT**
Cheung Sha Wan Government Offices
5th floor, 303 Cheung Sha Wan Road
Kowloon, Hong Kong

本署檔號 Our Ref. : AF GR CON 09/51
來函檔號 Your Ref. :
電話 Tel. No. : / For enquiries: 2150 6922
電郵地址 E-mail Address : mailbox@afcd.gov.hk
圖文傳真 Faxline No. : (852) 2311 3731

28 May 2013

**<u>Permission to Possess Traps for the Collection of Riparian Arthropods</u>**

The Director of Agriculture, Fisheries and Conservation has given permission to:

> **Yan-ling Elaine YUEN** of **the School of Biological Sciences, the University of Hong Kong** to possess Malaise traps, light traps, emergence traps, pan traps and suction samplers to collect riparian arthropods, <u>subject to the conditions on the reverse side of this permit</u>.

The permission is given in accordance with:

(a) Section 15 of the Wild Animals Protection Ordinance (Cap. 170)

(b) Regulation 18 of the Country Parks and Special Areas Regulations (Country Parks Ordinance (Cap. 208 sub. leg. A)

This permit expires on **30 June 2014**.

(Edmond LAM)
for Director of Agriculture, Fisheries and Conservation

Ms. Elaine YUEN,
M.Phil. candidate,
Lab 3N04, 3/F,
School of Biological Sciences,
Kadoorie Biological Sciences Building,
University of Hong Kong,
Pokfulam, Hong Kong.

## Conditions of Permission to Possess Traps for the Collection of Riparian Arthropods

1. This permission is limited to the possession of Malaise traps, light traps, emergence traps, pan traps and suction samplers to collect riparian arthropods in Hong Kong by Yan-ling Elaine YUEN of the School of Biological Sciences, the University of Hong Kong for scientific research purposes in accordance with the study proposal submitted to this Department in May 2013.
2. This permission does not exempt the permit holder from having to acquire any other necessary permission under the Laws of Hong Kong.
3. The permit holder shall only use Malaise traps, light traps, emergence traps, pan traps and suction samplers to collect riparian arthropods in local countryside and specified Country Parks (CP) and Special Areas (i.e. Ma On Shan CP, Lantau North CP, Sai Kung West CP, Shing Mun CP and Tai Po Kau Nature Reserve).
4. This permission does not authorize the entry to any leased land or licensed area or the collection or disturbance of the flora or fauna therein, in which case the prior approval of the lessees or the licence holders would be necessary.
5. This permission does not authorize the bringing of vehicles into Country Parks or Special Areas, in which case a permit in writing issued by the Country and Marine Parks Authority would be necessary.
6. The permit holder shall not affect in any way other permitted uses in Country Parks or Special Areas.
7. The permit holder shall label the traps and suction samplers in use with the name and telephone number of a contact person and shall inspect the traps regularly.
8. The permit holder shall release all non-target animals accidentally trapped immediately and hand over any Protected Wild Animals accidentally hurt by the traps and suction samplers deemed unsuitable for immediate release to this Department as soon as possible.
9. The permit holder shall only collect a minimal number of arthropod specimens at any one time.
10. The permit holder shall handle animals humanely and in a manner that will avoid their suffering.
11. The permit holder shall not offer the specimens collected to any other organization or person for commercial purposes.
12. The permit holder shall submit specimens collected for inspection upon request by any officer of this Department. One specimen of each of the arthropod species collected shall be sent to this Department upon request. For any new species that has not been described, an isotype or a paratype, if available, shall be sent to this Department once it is formally described.
13. The permit holder shall produce this permit for inspection on demand by any officer of this Department or police officer.
14. The permit holder shall provide a report on the quantity and species of specimens collected to this Department upon request.
15. A copy of each of the publications produced as a result of this study shall be sent to this Department for record.
16. The Director of Agriculture, Fisheries and Conservation reserves the right to recall or cancel this permission at any time without prior notification.

- END -

May 2013
Agriculture, Fisheries and Conservation Department
